# Mobile Technology for Improved Family Planning (MOTIF): the development of a mobile phone-based (mHealth) intervention to support post-abortion family planning (PAFP) in Cambodia

**DOI:** 10.1186/s12978-015-0112-x

**Published:** 2016-01-05

**Authors:** Chris Smith, Uk Vannak, Ly Sokhey, Thoai D. Ngo, Judy Gold, Caroline Free

**Affiliations:** 1 Department of Population Health, Faculty of Epidemiology and Population Health, London School of Hygiene and Tropical Medicine, Keppel Street, London, WC1 7HT UK; 2 Marie Stopes International, Phnom Penh, Cambodia; 3 Research, Monitoring and Evaluation Team, Marie Stopes International, London, UK; 4 Independant Consultant, London, UK

**Keywords:** Contraception, Family planning, Post-abortion family planning, mHealth, Mobile technology

## Abstract

**Background:**

The objective of this paper is to outline the formative research process used to develop the MOTIF mobile phone-based (mHealth) intervention to support post-abortion family planning in Cambodia.

**Methods:**

The formative research process involved literature reviews, interviews and focus group discussions with clients, and consultation with clinicians and organisations implementing mHealth activities in Cambodia. This process led to the development of a conceptual framework and the intervention.

**Results:**

Key findings from the formative research included identification of the main reasons for non-use of contraception and patterns of mobile phone use in Cambodia. We drew on components of existing interventions and behaviour change theory to develop a conceptual framework. A multi-faceted voice-based intervention was designed to address health concerns and other key determinants of contraception use.

**Conclusions:**

Formative research was essential in order to develop an appropriate mHealth intervention to support post-abortion contraception in Cambodia. Each component of the formative research contributed to the final intervention design.

**Electronic supplementary material:**

The online version of this article (doi:10.1186/s12978-015-0112-x) contains supplementary material, which is available to authorized users.

## Background

Cambodia is one of the poorest and least developed countries in Asia. Eighty percent of Cambodians live in rural areas, 45 % are under the age of 20 and approximately 28 % live below the poverty line. Despite recent improvements in health indicators, maternal mortality remains high at 206 deaths/100,000 live births [1]. Although the total fertility rate declined to 3.0 births per woman in recent years, there remains unmet need for contraception. The 2010 Cambodia Demographic and Health survey (DHS) reported that although 81 % of women of reproductive age wanted to delay their next child or have no more children, only 35 % reported using a modern contraceptive method [1].

The abortion rate in Cambodia has been estimated to be 50/1,000 women, compared to the global average of 28/1,000 [2]. As fertility can return within two weeks it is important to start contraception as soon as possible after abortion if the woman wants to prevent subsequent unintended pregnancies [3]. In Cambodia, amongst women who have had an abortion, 26 % have had more than one [1]. This suggests there is a need to improve contraception uptake and continuation post-abortion.

Mobile phone-based interventions (‘mHealth’) refer to the use of mobile technologies for health [4]. mHealth interventions can utilise different modes of communication; for example, text-message, voice messages, video and smartphone applications and may involve one-direction or two-way (interactive) communication [5]. mHealth interventions can deliver support inexpensively wherever a person is located, whenever it is needed. In particular, mHealth has the potential to reach youth and rural populations, where geographical distances can restrict access to in-person services [6].

Although a number of mHealth contraception initiatives have been launched and scaled up in low-income settings, to date, the effect of mHealth interventions on post-abortion family planning (PAFP) have not been reliably established. There is therefore a need for more evidence on the effectiveness of mHealth interventions for PAFP.

Formative research is often used to design context-specific interventions. It may involve literature reviews and research with the target population and other key stakeholders [7]. However, the process is rarely reported [8]. This paper outlines the formative research process we used to inform development of the MOTIF intervention to support PAFP in Cambodia.

## Methods

The MOTIF study was conducted at four Marie Stopes International Cambodia (MSIC) clinics providing comprehensive sexual and reproductive healthcare services; two serving predominantly urban populations around Phnom Penh City, and two serving predominantly rural populations (Battambang and Siem Reap). Ethical approval was granted by the LSHTM ethics committee, the MSI ethics committee and the Cambodia Human Research.

### Ethics Committee

Development of the MOTIF intervention was iterative and included processes recommended for the development of complex interventions [9]. We drew on existing knowledge and reviewed literature (using simple keyword searches) in the following areas with particular reference to Cambodia; determinants of contraceptive use, behaviour change theory, interventions delivered by mobile phone in other areas, interventions for improving contraception use.

Research conducted in Cambodia included a case note review of clinic data, interviews and focus group discussions (FGD), consultation with clinicians and local organisations implementing mHealth activities, development of a conceptual framework, and testing the intervention with the target group.

Case note review of 100 MSIC abortion clients was conducted during October and November 2012 using routinely collected data to estimate baseline event rates. We identified the first 25 clients seeking abortion services at each of the four clinics attending sequentially from 1st September 2011 from the clinic register. Repeat attendances over subsequent 12-months were identified. Where available, data was collected on age of client, residence, provision of a mobile phone number, contraceptive history, reason for abortion, subsequent follow-up attendances, contraceptive use and abortions. Data were summarised using simple statistics. Additional file 1 contains a summary of characteristics of clients from the case note review, interviews and FGDs.

Following the case note review we conducted interviews and FGDs with clients seeking abortion services. Clients attending for abortion services were recruited sequentially, and asked if they would like to participate at the end of their post-abortion counselling session. Clients were provided with an information sheet to read, or it was read to them, and provided signed or thumb-printed consent. Clients were either interviewed at the time of attending for abortion services or when attending for follow-up appointment, according to their preference.

Author UV conducted 15 semi-structured interviews with clients during December 2013 and January 2014 across the four study clinics in the Khmer (Cambodian) language. A topic guide was developed containing questions to explore clients’ reasons for seeking abortion services, contraception and mobile phone use and to seek views on the proposed intervention (see Additional file 1). Clients were contacted for a follow-up phone interview after one-month. There were three refusals because clients stated that they didn’t have time. Interviews were recorded and transcribed into English and read by author CS to identify key themes.

Four FGDs were conducted during January and February 2014; two at urban clinics, and two at rural clinics. The aim of the FGDs was to test proposed messages. The FGD topic guide was informed by the interviews. FGDs were conducted by authors CS and UV and a summary of the discussion transcribed to English. Transcripts were read by CS to identify key themes. These findings were used to guide intervention development. We also consulted with other organisations implementing mobile phone-based interventions in Cambodia.

We developed a preliminary intervention based on these activities. The messages were then tested with eleven interview and FGD participants who agreed to receive messages on their mobile phones. Messages were modified based on feedback received on content, tone, speed of voice, and sound quality.

## Results

### Literature on determinants of contraceptive use, with a focus on Cambodia

We reviewed literature on determinants of contraceptive use in order to identify areas amenable to an intervention. We found Sheeran’s framework to be useful as it combines developmental models (describing contraceptive use resulting from transition through a series of stages) and decision-making models (psychological factors that speed up or delay this transition). Sheeran’s framework considers contraceptive use as a product of *background*, *intrapersonal*, *interpersonal* and *situational* factors [10]. The 2010 Cambodia DHS collected data on *background* factors associated with contraceptive use reporting increased contraceptive use with increasing parity, wealth, and levels of educational attainment [1]. Westoff conducted additional analysis on *intrapersonal*, *interpersonal* and *situational* reasons for non-use of contraception [11]. Globally, health concerns including side-effects are the commonest reasons for non-use of contraception; this is particularly evident in Cambodia, with health concerns accounting for almost 50 % of non-use [11, 12].

We identified qualitative research supporting these findings. Experience of negative side-effects may be related to traditional health beliefs in Cambodia [13]. Petitet and Desclaux reported that in Cambodia, abundant menstruation (the expulsion of ‘bad blood’) is viewed as a sign of good health and fertility. Contraceptive methods that cause reduced menstruation can cause women to worry about blood retention leading to discontinuation.

Dingle identified factors related to knowledge, access, cost and autonomy as important reasons for non-use of contraception in Cambodia. Women often obtain information about contraception from friends and relatives, with myths and rumours commonplace [14]. Furthermore, not all women have full autonomy over health care decision-making. The Cambodia DHS reported that only 45 % of married women make their own decisions about health care [1]. Sex workers in Cambodia have even less autonomy, often reporting coercion by clients into unprotected sex [15]. Finally, access and cost can influence choice of contraceptive method, particularly for poor women living in rural areas [14].

### Behaviour change theory

Lopez highlighted the need for increased attention to theory of behaviour change in designing and evaluating interventions for contraception use [16]. In addition to understanding existing contraceptive behaviour, we drew on behaviour *change theory.* Michie defined ‘behaviour change interventions’ as *‘coordinated sets of activities designed to change specified behaviour’* and developed a taxonomy of behaviour change techniques, e.g., provision of information on risks, consequences of actions, or conversely, encouragement and goal setting [17]. We applied this framework as its development involved a systematic literature review, evaluation of existing frameworks and reliability testing. At its core a ‘behaviour system’ involves three essential conditions; *capability* [an individual’s psychological and physical capacity to engage in the activity), *motivation* (the brain processes that direct behaviour which include conscious decision-making and emotional responding) and *opportunity* (factors that lie outside the individual that make behaviour possible) [17].

### Literature on interventions delivered by mobile phone in other areas

We reviewed evidence from systematic reviews of mHealth interventions. Free, et al.,’s systematic review of mHealth interventions found that there is currently no evidence of benefit for simple text-message medication reminders (RR 1.00, 0.77–1.30) [6]. This is consistent with existing adherence research demonstrating that multifaceted interventions can be effective but uni-faceted interventions have modest benefits [18].

Multi-faceted mHealth interventions have been shown to increase smoking cessation in a high-income setting and adherence to HIV medication in a low-income setting [19, 20]. Of particular interest was the WellTel trial in Kenya, evaluating a multi-faceted intervention for HIV medication adherence [21]. Participants were sent a weekly text-message in the local language asking, *“How are you?”* If the message was seen by a third person it was not obvious that that it was sent from a HIV clinic. Health workers would then phone clients who reported a problem. Medication adherence was higher in the intervention group, demonstrating that an mHealth intervention could be effective when confidentiality and privacy are important.

### Literature on mHealth in Cambodia

We found limited published literature on mHealth in Cambodia although Cambodians are enthusiastic mobile phone users, as can be witnessed during daily life in both urban and rural areas. Although mobile phone ownership is estimated to be over 90 %, most Cambodians use simple rather than internet-enabled smartphones [22]. There is significant interest in mHealth in Cambodia among an increasing number of organisations.

Bullen identified a number of operational challenges facing mHealth programmes in Cambodia [23]. Cambodian mobile phone users often have multiple Subscriber Identification Module (SIM) cards and share phones, which can make it difficult to maintain contact with users. Cambodians often prefer to use their mobile phone for voice calls rather than text-messages as many phones lack Khmer language capability. Furthermore, whilst literacy levels are 90 % in urban areas, this figure is lower (69 %) in rural areas [1].

### Literature on interventions for contraception including those delivered by mobile phone

We next reviewed literature on behaviour change interventions to increase contraception use. Systematic reviews have found limited evidence for interventions to improve contraception and PAFP [24, 25]. We identified three trials of mHealth interventions aimed at increasing use of contraception, all evaluating text-message interventions. The two trials that did not show an effect both evaluated simple uni-faceted text-message interventions (contraception reminders) [26, 27]. The intervention that was effective comprised a variety of daily educational text-messages and oral contraceptive (OC) reminders [28].

Other successful mHealth contraception initiatives in low-income settings include Mobile for Reproductive Health (m4RH) and Mobile Alliance for Maternal Action (MAMA). m4RH used best practices from health communication programs to systemically develop family planning text messages and MAMA developed adaptable messages based on WHO and UNICEF guidelines [29].

### Findings from the case note review

The case note review found uptake of effective PAFP either immediately after abortion or at two-week follow-up to be 40 % (*n* = 40) (OC, intra-uterine device, implant, injection). Over 50 % of clients did not return to the MSIC clinic for any reason within 12-months of having an abortion. 4 % of clients returned to the same clinic for repeat abortion within 12-months with 8 % returning with repeat pregnancy. Over 80 % of clients provided a mobile phone number.

### Findings from the interviews and FGDs (Table 1)

**Table 1:** Quotes from interviews and focus group discussions with clients

Related to current abortion
*“If we want to have more children, those who go to school must drop out because we have no enshrined money for their study”* (age 30, married, two children)
*“We do not have enough money yet…my husband stays far away from me…he always goes to province”* (age 24, married, no children)
*“I discussed with my husband. He said just do what I want to do”* (aged 34, married, one child)
Reported previous experience with contraception use
*“I wasn’t using it regularly so I got sick because of it…it felt hot inside my chest and I felt exhausted… Thus I changed to condom but difficulty is it enables cervicitis”* (aged 34, married, one child, talking about previous experience with OC)
*“Because we feel so tired after coming back from the business and we don’t take it regularly or maybe we forget to take it one evening, so we’re lazy”* (aged 30, married, two children talking about previous experience with OC)
*“Husband heard from a friend that ‘when we use condom a girl can be burned, it is not good for both husband and wife’. So I followed my husband”* (aged 24, married, no children, talking about previous contraception use)
Reported factors influencing use of post-abortion family planning
*“I am not able to afford any of these methods to prevent pregnancy. If I could afford I would practice the contraceptive method…I think I might wait for my monthly salary”* (aged 28, separated, no children, talking about PAFP)
*“Not interested in contraception yet…because my health is not so good”* (aged 21, married, talking about plans for PAFP)
*“She told me a lot but I forgot some because there’re a lot of methods”* (aged 26, married, no children, talking about PAFP counselling received)
Reported mobile phone use
*“Even when the company sends messages we can’t read and leave alone the messages sent”* (aged 30, married, two children)
*“I don’t really understand the message in the phone”* (aged 34, married, one child)
*“My older sister sent a message and I got my husband to read it”* (aged 30, married, two children)
*“Husband pays bill but never picks up my phone to answer”* (aged 26, married)
Views on the intervention
*“I think its good because we need contraceptive method to prevent pregnancy, so we need some advice to do this or that”* (aged 31, married, no children)
*“Such as service is really good…for women and their health and there can be a lot of side-effects if they have frequent abortions…it means they take care of us”* (aged 21, married)
*“We talk on phone, no-one knows our face…If anyone said that they saw us drive here, they would think that we didn’t come here to discuss but to do something, so if we have this programme I think that its very good…it makes clients reduce the time to come directly”* (aged 26, married, no children)
*“We are ignorant and cannot read the messages so we leave them we see them, so I suggest talking directly with each other”* (aged 30, married, two children)

Interview and FDG participants reported seeking abortion services for mainly economic, or less commonly, health reasons. Most women disclosed the abortion to their husband or partner. Many women reported side-effects when using contraception in the past. Clients reported it was difficult to make PAFP choices at the time of abortion. Reasons for this included wanting to wait for their health to improve first, having to wait for their salary or needing to discuss with their husband. Some women reported they were unable to retain sufficient information about contraception. When we contacted clients for the one-month post-interview phone call they often had many questions relating to contraception and side-effects.

Regarding the proposed mHealth intervention, most clients said they preferred to make phone calls over text-message. Most could not read and many had never used text-messaging. However, most reported listening to voice messages received on their phones. All clients were positive about the proposed new MOTIF intervention. Despite sharing of phones, privacy was not mentioned as a particular concern. Most clients stated that messages sent to their phones should mention the terms ‘Marie Stopes’ and ‘contraception’ so they would know the topic of the message and who it was from. Consultation with clinic staff and organisations implementing mHealth activities in Cambodia also suggested a limited likelihood of success with a text-message intervention.

### Developing a conceptual framework and the final intervention

**Fig. 1:**
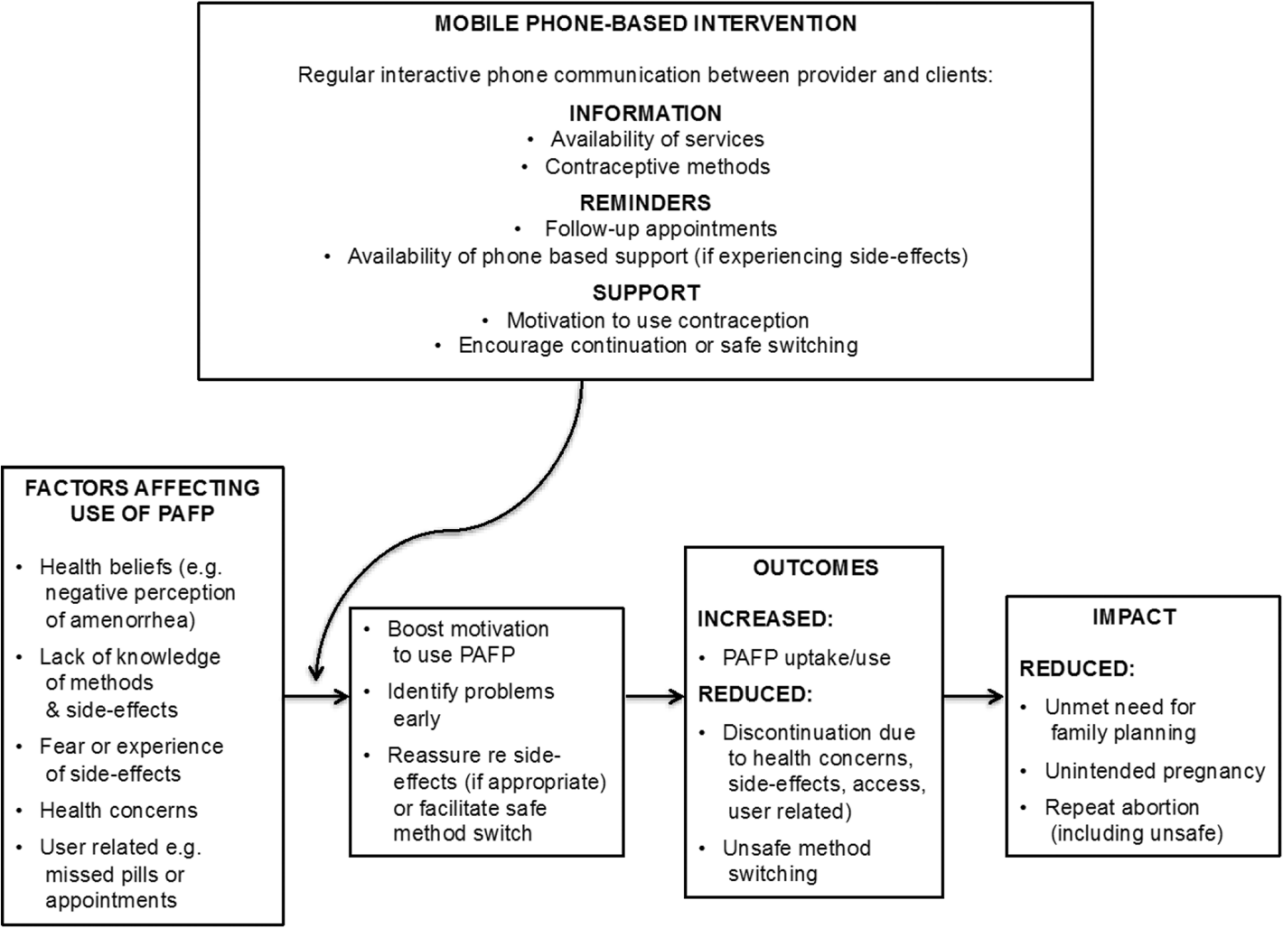
Conceptual Framework for the MOTIF intervention in Cambodia

We developed a conceptual framework (Fig. 1) for the intervention that considered determinants of contraceptive use, links between contraceptive use and fertility and theory of behaviour change. There is an emphasis on addressing *intrapersonal* factors, in particular health concerns. The intervention aimed to improve clients *capability* to use contraception by providing information about methods, *opportunity* to use contraception i.e. informing clients where they can access contraception near to where they live, and *motivation* by re-enforcing the benefits of contraception use and providing support relating to side-effects [17].

We tested the messages with clients and made final modifications based on their feedback. See Table 2 for a description of the final intervention. Table 3 summarises how formative research findings contributed towards the intervention design.

**Table 2:** Final MOTIF intervention

The MOTIF final intervention comprised a series of automated voice messages to participants’ mobile phones over the three-month period following their abortion, at the time of day of their preference. Clients received the first message within one-week of receiving abortion services and then every two-weeks, with a total of six messages. The main message, recorded in the Khmer language, was as follows:
*‘Hello, this is a voice message from a Marie Stopes counsellor. I hope you are doing fine. Contraceptive methods are an effective and safe way to prevent unplanned pregnancy. I am waiting to provide free and confidential contraceptive support to you. Press 1 if you would like me to call you back to discuss contraception. Press 2 if you are comfortable with using contraception and you do not need me to call you back this time. Press 3 if you would prefer not to receive any messages again’*
Clients who pressed 1, or who did not respond to the message prompts, received a phone call from a counsellor. The phone calls aimed to support contraceptive use by addressing clients’ *capability* to use contraception by providing individualised information on a range of contraceptive methods, *opportunity* to use contraception e.g. informing clients where they could access specific methods near to their residence, and *motivation* by re-enforcing the benefits of contraception use. If the client requested, the counsellor would also discuss contraception with the husband or partner. Follow-up calls to clients were made during preferred times indicated by the client on her registration form. Clients were also able to call the MOTIF service at any time to request to speak with a counsellor. Clients that chose to receive the OC or injectable could opt to receive additional reminder messages appropriate to their method (e.g. to start a new packet of pills or when to receive a new injection). The sixth and final voice message provided similar information to the first five, but also reminded the client that this would be the last message they will receive. The MOTIF intervention was delivered by trained counsellors at the MSIC head office in Phnom Penh. Voice messages were scheduled and sent using the open-source software programme ‘Verboice’, developed by InSTEDD (instedd.org). MSIC incurred the cost of outgoing communication from the provider to client, and clients incurred any costs calling into the service (the cost of a local call).

**Table 3:** Implications of key formative research findings for intervention design

Formative research component	Key findings	Implication for intervention design
Insights from contraception literature	• Health concerns identified as major reason for non-use. Other reasons include factors related to access, cost, autonomy	• Intervention needs to address health concerns as well as factors related to access, cost (by nforming clients where they can access contraception near their home) and autonomy
	• Limited evidence for interventions to improve adherence to specific contraceptive methods or uptake of PAFP	• The intervention needs to anticipate some discontinuation and aim to facilitate safe method switching and well as support continuation with existing method
	• Most discontinuation occurs within the first few months	• Decided to provide intervention for three-months
Insights from mHealth intervention and behaviour change literature	•Uni-facteted* adherence interventions have at best modest effects	• Developed a multi-faceted intervention providing information reminders and support to boost motivation to use PAFP
	• A semi-automated mHealth intervention increased adherence to HIV treatment in Kenya	• A similar intervention could be adapted for PAFP in Cambodia
Case note review	• 40 % uptake of effective PAFP at the time of seeking abortion services	• An mHealth intervention is an opportunity to maintain contact with clients that don’t return to the clinic for contraception after seeking abortion services
	•Over 50 % clients did not return to the clinic within 12-months	
Interviews	• Side-effects with contraception common	• Re-enforced findings from literature that intervention should address health concerns
	• Clients can find it difficult to make decisions about PAFP at time of seeking abortion services	• The mHealth intervention is an opportunity to maintain contact and remind clients about available methods
	• Women sometimes have to discuss with their husband/partner before using contraception	• Re-enforced findings from literature review that the intervention take into account women’s lack of autonomy, facilitating a discussion with husband/partner if appropriate
Focus group discussions	• Preference for voice rather than text-based intervention	• Intervention used voice messages sent to clients phone instead of text-messages
	• Many clients preferred direct phone call to automated message	• Developed a semi-automated intervention as fully counsellor delivered intervention would be costly to scale up
	• Clients preferred that the messages mentioned the terms ‘Marie Stopes’ and ‘contraception’	• Voice message mentioned ‘contraception’ and ‘Marie Stopes’, but not the name of the client
Consultation with MSIC staff and other organisations	• Text-message interventions likely to have limited success in Cambodia	• Re-enforced findings from clients that intervention should use voice rather than text
	• A fully counsellor delivered intervention would be costly and hence harder to scale-up	• Intervention was semi-automated aiming to identify clients most in need of additional support

*A unifaceted interventions refers to single-component intervention. A multi-faceted intervention refers to a complex intervention using a range of behaviour change techniques

## Discussion and conclusions

We used a wide range of methods to inform the development of the MOTIF intervention. Our literature review indicated that our intervention should address intrapersonal factors, in particular health concerns. We found limited evidence on behaviour change interventions to increase the use of specific contraceptives. Whilst this may be partly due to a limited number of high quality, adequately powered trials, the focus of the interventions needs to be examined. Interventions often focus on adherence to a specific method, which may be less effective than an intervention that anticipates method-specific discontinuation and facilitates safe method switching [12]. We designed an intervention to offer to all women seeking abortion services, regardless of the immediate plans for PAFP.

A key finding from the case note review was high unmet need for PAFP. It was not known if clients used other providers to obtain contraception as most women did not return to MSIC clinics. Most clients provided a mobile phone number, which led us to believe that a mHealth intervention could be an opportunity to maintain contact with clients. Interviews and FGDs supported these findings. Health concerns related to contraception were widely reported. Clients also stated a clear preference for a voice-based intervention. In developing the conceptual framework and intervention we hypothesised that a multi-faceted mHealth intervention would remind clients about contraceptive methods, identify problems with side-effects early, provide support, and boost motivation to use PAFP, while reducing discontinuation and method switching.

The strength of this paper is that we have clearly described how each component of the formative research contributed to the final intervention design. Interventions are often developed without evidence of having gone through a formative research process or without clearly describing that process. Furthermore, existing behaviour or theoretically predicted mechanisms of action are not fully described [8, 17]. Our research allowed us to gain an understanding of successful interventions, and to avoid repeating mistakes of unsuccessful projects. Our limitations were mainly due to time and resource constraints. A limitation of our formative research is that analysis of the interviews and FGDs was not undertaken by a second coder. Furthermore, the literature searches were not systematic. However, it is important to consider when to stop the development process [8].

Using a similar approach to Lester, et al., we developed a semi-automated intervention, which sought to identify clients most in-need of counsellor delivered support [21]. Where the MOTIF intervention differs from Lester and other mHealth contraception interventions is that it is voice-based. The main reasons for this were rural literacy levels, lack of functionality of Khmer script on phones, and client preference. This study provides some insights into mobile phone-based interventions intervention development in low-literacy settings.

The MOTIF intervention was associated with an increase in self-reported contraception use four months post-abortion but not at 12 months. The intervention was associated with increased long-acting contraception use at four and 12 months [30, 31]. A process evaluation will explore participants’ experience and cost comparison of MOTIF with other interventions.

### Ethics

Ethical approval was granted by the LSHTM ethics committee, the MSI ethics committee and the Cambodia Human Research Ethics Committee for both the formative research (Phase 1) and the trial (Phase 2). The MOTIF trial is registered with ClinicalTrials.gov, number: NCT01823861.

## Additional file

Additional file 1: **Supplementary material.** (DOC 182 kb)

## References

[1] National Institute of Statistics, Directorate General for Health and IM. Cambodia Demographic and Health Survey 2010 [Internet]. Health (San Francisco). 2010. Available from: http://www.measuredhs.com/pubs/pdf/FR249/FR249.pdf.

[2] Fetters T, Samandari G (2009). An estimate of safe and unsafely induced abortion in Cambodia.

[3] World Health Organization Department of Reproductive Health and Research (WHO/RHR) and Johns Hopkins Bloomberg School of Public Health/Centre for Communications Programs (CCP) Knowledge for Health Project (2011). Family Planning: A Global Handbook for Providers (2011 update).

[4] Mechael P, Batavia H, Kaonga N, Searle S, Kwan A, Goldberger A, et al. Barriers and Gaps Affecting mHealth in Low and Middle Income Countries: Policy White Paper. Centre for Global Health and Economic Development. Earth Institute, Columbia University, USA; 2010.

[5] Källander K, Tibenderana J, Akpogheneta O, Strachan D, Hill Z, Ten Asbroek A (2013). Mobile Health (mHealth) Approaches and Lessons for Increased Performance and Retention of Community Health Workers in Low- and Middle-Income Countries: A Review. J Med Internet Res.

[6] Free C, Phillips G, Galli L, Watson L, Felix L, Edwards P (2013). The effectiveness of mobile-health technology-based health behaviour change or disease management interventions for health care consumers: a systematic review. PLoS Med.

[7] Newes-Adeyi G, Helitzer DL, Caulfield LE, Bronner Y (2000). Theory and practice: applying the ecological model to formative research for a WIC training program in New York State. Health Educ Res.

[8] Hill Z, Manu A, Tawiah-Agyemang C, Gyan T, Turner K, Weobong B (2008). How did formative research inform the development of a home-based neonatal care intervention in rural Ghana?. J Perinatol.

[9] Craig P, Dieppe P, Macintyre S, Michie S, Nazareth I, Petticrew M. Developing and evaluating complex interventions: new guidance [Internet]. Sciences-New York. 2008. Available from: www.mrc.ac.uk/complexinterventionsguidance. 10.1136/bmj.a1655 PMC2769032 18824488

[10] Ogden J. Health Psychology: A textbook. 5th ed. Open University Press, UK; 2012.

[11] Westoff C (2012). Unmet Need for Modern Contraceptive Methods: DHS Analytical Studies No. 28.

[12] Ali M, Cleland J (2010). Contraceptive switching after method-related discontinuation: levels and differentials. Stud Fam Plann.

[13] Petitet P, Desclaux A. Reproductive health and HIV in Cambodia: from anthropology to public health [Internet]. Groupe de Recherche Cultures, Santé, Sociétés, Paul Cézanne Université, Aix Marseille III (France); 2011. Available from: http://hal.archives-ouvertes.fr/docs/00/58/42/95/PDF/Hancart_Desclaux_2010.pdf.

[14] Dingle A. Exploring experiences of and reasons for use and non-use of reproductive and maternal health vouchers among targeted beneficiaries, programme implementers and service providers in the Vouchers for Reproductive Health Services (VRHS) programme: Preliminary. London School of Hygiene and Tropical Medicine, UK; 2012.

[15] Morineau G, Neilsen G, Heng S, Phimpachan C, Mustikawati D (2011). Falling through the cracks: contraceptive needs of female sex workers in Cambodia and Laos. Contraception.

[16] Lopez L, Tolley E, Grimes D, Chen-Mok M (2011). Theory-based interventions for contraception (Review). Cochrane Database Syst Rev.

[17] Michie S, van Stralen MM, West R (2011). The behaviour change wheel: a new method for characterising and designing behaviour change interventions. Implement Sci BioMed Central Ltd.

[18] Haynes R, Ackloo E, Sahota N, McDonald H, Yao X (2008). Interventions for enhancing medication adherence (Review). Cochrane Database Syst Rev.

[19] Horvath T, Azman H, Kennedy G, Rutherford G (2012). Mobile phone text messaging for promoting adherence to antiretroviral therapy in patients with HIV infection (Review). Cochrane Database Syst Rev.

[20] Free C, Knight R, Robertson S, Whittaker R, Edwards P, Zhou W (2011). Smoking cessation support delivered via mobile phone text messaging (txt2stop): a single-blind, randomised trial. Lancet.

[21] Lester R, Ritvo P, Mills E, Kariri A, Karanja S, Chung M (2010). Effects of a mobile phone short message service on antiretroviral treatment adherence in Kenya (WelTel Kenya1): a randomised trial. Lancet.

[22] Kimchhoy P, Sareth U, Sola J. Research Report on Existence and Use of Phones that permit written communication in Khmer Script [Internet]. Open Institute; 2013. Available from: http://www.open.org.kh/en.

[23] Bullen P (2013). Operational Challenges in the Cambodian mHealth revolvution. J Mob Technol Med [Internet].

[24] Halpern V, Lopez L, Grimes D, Stockton L, Gallo M (2013). Strategies to improve adherence and acceptability of hormonal methods of contraception (Review). Cochrane Database Syst Rev.

[25] Tripney J, Kwan I, Bird K (2013). Postabortion family planning counseling and services for women in low-income countries: a systematic review. Contraception.

[26] Hou M, Hurwitz S, Kavanagh E, Fortin J, Goldberg A (2010). Using daily text-message reminders to improve adherence with oral contraceptives: a randomized controlled trial. Obstet Gynecol.

[27] Tsur L, Kozer E, Berkovitch M (2008). The effect of drug consultation center guidance on contraceptive use among women using isotretinoin: a randomized, controlled study. J Women’s Heal.

[28] Castano P, Bynum J, Andres R, Lara M, Westhoff C (2012). Effect of daily text messages on oral contraceptive continuation; a randomised controlled trial. Obstet Gynecol.

[29] L’engle K, Vahdat H, Ndakidemi E, Lasway C, Zan T (2013). Evaluating feasibility, reach and potential impact of a text message family planning information service in Tanzania. Contraception. Elsevier Inc.

[30] Smith C, Vannak U, Sokhey L, Ngo TD, Gold J, Khut K (2013). MObile Technology for Improved Family Planning services (MOTIF): study protocol for a randomised controlled trial. Trials [Internet].

[31] Smith C, Ngo TD, Gold J, Edwards P, Vannak U, Machiyama K, et al. Effect of a mobile phone-based intervention on post-abortion contraception: a randomized controlled trial in Cambodia. Bull World Health Organ [Internet]. 2015; Available from: http://www.who.int/bulletin/volumes/93/12/15-160267/en/. 10.2471/BLT.15.160267 PMC4669734 26668436

