# Supplementary material for: Mobile Technology for Improved Family Planning (MOTIF): the development of a mobile phone-based (mHealth) intervention to support post-abortion family planning (PAFP) in Cambodia
Source: Reprod Health. 2016 Jan 5;13:1. doi: 10.1186/s12978-015-0112-x (PMC4700587; doi:10.1186/s12978-015-0112-x)
Supplement: Supplementary file 1 — Supplementary material. (DOC 182 kb) [file 12978_2015_112_MOESM1_ESM.doc]

| **Interview topic guide**  *Demographics:* Questions about age, residence, distance from clinic, literacy  *1) Views on current service*   - You have recently used the Marie Stopes services. Could you tell me about your experience of the   services?   - What information did they give you about contraception? - What did you think about any advice they offered about contraception?  *Prompt for:* any counselling offered, acceptable tone, content of information, relevant? What method did you use? What different methods of contraception did the staff inform you about? Were the staff able to answer all of your questions? Did the staff arrange follow up? - Have you used the Hotline service at MSI?   *If yes:* could you tell me about your experience of using this service?  *Prompt for:* If you experienced unexpected effect’s from contraception what advice were you given? Any comments about privacy and confidentiality when discussing contraception over the phone? Do you have any suggestions for improvement?  *If no:* any reason for this  *2) Contraception use*   - Have you ever used contraception?   If yes: when was the last time you used contraception? Which method? What was your experience of taking contraception? *If discontinued:* Why did you stop it? Any unexpected- effects? Did you seek advice, if so from whom? Have you ever used other methods of contraception? *(if yes, repeat above questions)*  *If using contraception when became pregnant:* Why do you think you became pregnant even whilst you were using contraception? (probe for missed pills diarrhoea, or other cause of contraceptive failure) Where would you obtain contraception usually? Motivation for using contraception?  If no: any reason for this?   - What are your current plans for contraception? Ideally, where would you go to get contraception? *Probe* *on* community based, fixed clinic, private provider *and* why? Do you have any children? How many? Desired family size?   *3) Reasons for abortion*  If not already raised:   - When did you realise you were pregnant? Had you wanted to be pregnant?   *Prompt for:* Reasons for abortion (e.g. medical, social)? Discussed with others?  *4) Views on the intervention*  • What do you think about receiving text messages or phone calls relating to contraception? *Prompt for:*  preference for phone/text, types of message, frequency, language  *Mobile phone usage*  • Do you have access to a mobile phone?  *If yes: Prompt for:* Network, type of phone, Smartphone. Do you own the phone?   - How do you use your phone? *Prompt for:* SMS/phone call/internet/preferences   *If no: Prompt for:* Any reasons why not? Any future plans to get phone?  Can I finally ask you for any final comments that have not been covered in this interview. Thank you very much for helping us and giving up your time  *One-month follow-up phone call*   - How are you? Is this a suitable time for you to talk? - Are you currently using a contraceptive method?   *If yes:*  which method? How are you taking it? Any problems?  *If no:*any reason for this? *If a method discontinued:* Why did you stop it? Any unexpected- effects? Did you seek advice, if so from whom?  • What are your future plans for contraception?  Do you have any other comments? Thank you very much for helping us and giving up your time  **FGD topic guide**  *Mobile phone use*   - Do you have a mobile phone? Is it your own phone or shared? Who pays for credit? - How do you use your phone? Preference for SMS vs. Phone (inc.voicemail) - Literacy: read or write Khmer or English? - Privacy/confidentiality: does anyone else have access to your phone (either answer the phone or check SMS messages)? Is this a problem? Are you able to make calls in private?   *PAFP*   - Current/future plans for contraception and pregnancy. Reasons for use/non-use; where do you plan to go to get contraception in the future? - Disclosure to others about contraception use (?also abortion); reasons   *Views on the proposed new service*   - Would you be interested in receiving a service provided by MSI to provide support via your mobile phone for contraception after abortion? - Do you have any suggestions for this service? - Any comments if we were sending you sensitive messages or discussion about contraception over the phone? Would this be a problem?   *If relevant:* would you still be interested in the service even if you haven’t decided to use contraception at the current time?  *Scenarios*  (1) Regular communication from MSI to client to check for any problems or provide info   - Comments on this - SMS or phone? - Automated message vs. having a ‘real time’ conversation - If a pre-recorded voice message (or SMS) do you have any suggestions as to what  the message could say?   If no suggestions ask for feedback on the following 3 messages and which  one they prefer:   1. “Hello, how are you? This is a message from Marie Stopes to check  how you are doing. Press 1 if you are fine, or 2 if you have a problem and we will call you back” 2. “Hello, how are you? This is a message from Marie Stopes to check  how you are doing. If you have any questions for us please leave a message after the beep and we will respond to you with an automatic message within 2 days” 3. “Hello, how are you? This is a message from Marie Stopes to check how you are with regards to contraception. Press 1 if you are fine, or 2 if you have a problem.” If client presses 2, then the client receives a series of options asking what the problem is 1,2,3,4,5 etc. to identify and provide a response straight away  - Any comments on whether we should mention ‘Marie Stopes’ or ‘contraception’ or your name in the message? - If a VOICE message asked for a response e.g. Press 1 if you are fine, or 2 if you have a problem, would you be able to do that? Would you be able to leave a voice message so that we can respond later? - If a simple SMS (e.g. OK?) that required the client to press 1 if you are fine, or 2 if you have a problem, would you be able to do that? - Comments on the frequency of messages to clients?   (2) Side-effect from contraception (example irregular bleeding, headache from pill or depot)   - What would you do? Would you consider phoning the new service for advice? What  if this involved the cost of a local call? (explain this might make the service more  ‘sustainable’) - Views on automated response (and able to leave a message) vs. having a ‘real time’  conversation (explain automated might also make the service more ‘sustainable’) - Views on instant response vs. delay of 1-2 days - How about if we linked you up with another client using a method to share experience? Would you mind sharing experience as a ‘model client’?     (3) Daily pill reminder (additional service if using pill)   - Opinions on this; SMS vs. phone. What could that message say? (e.g. *OK PILL*) - Or for calendar method?   (4) Appointment reminder   - Opinions on this; SMS vs. phone. What could that message say? (e.g. *Marie Stopes appointment 1 week*)   (5) Problem after abortion e.g. bleeding or pain   - Opinion on phone call or SMS a few days or a week after abortion to ask how the client is after abortion (as well as asking about contraception)   (6) Other scenarios?   - Any other suggestions? Any other information that clients would like to receive via  mobile phone? - Based on our discussion about how we might provide the service, how long do you think we should provide this service to clients after they have an abortion?   *Summing up*   - Summarise key points raised in the FGD - Ask if anything has been missed or participants want to add anything |
| --- |

Supplementary material: Interview and focus group discussion topic guides

| | **Characteristics of 100 case note review clients** | | | | --- | --- | --- | | **Age (years)** | Average | 29.3 | |  | Range | 18-46 | |  | <25 | 28 (28%) | |  | 25 or above | 72% | | **Employment status*** | Employed | 10 (10%) | |  | Factory worker | 22 (22%) | |  | Self-employed | 12 (12%) | |  | Farmer | 23 (23%) | |  | Entertainment worker | 2 (2%) | |  | Housewife | 31 (31%) | | **Martial status**** | Married | 100 (100%) | | *Self-reported employment status according to MSIC client registration form. Categories may not be mutually exclusive  **It is possible that women do not disclose being single given that pregnancy outside of marriage is not socially acceptable in Cambodia | | |  | **Characteristics of focus group discussion (FGD) participants** | | | | --- | --- | --- | | **FDG** | **Location** | **Participants** | | 1 | Urban | Two participants. Limited data on clients that agreed but then did not join | | 2 | Urban | Three participants. Conducted on a Sunday. Possible that more factory workers may have attended this group | | 3 | Rural | Six participants: all mainly users of PAFP and older women | | 4 | Rural | Five participants: non-users of PAFP and younger if compared to CBB |  | **Characteristics of interview participants** | | | | | | --- | --- | --- | --- | --- | | **No.** | **Age** | **Occupation** | **Marital status** | **Clinic** | | 1 | 20 | Factory worker | Married | Urban | | 2 | 32 | Business | Married | Urban | | 3 | 21 | Company staff | Married | Urban | | 4 | 26 | NGO staff | Married | Rural | | 5 | 26 | Housewife | Married | Rural | | 6 | 26 | Business (sells clothes) | Married | Rural | | 7 | 30 | Business | Married | Rural | | 8 | 36 | Farmer-migrant | Separated | Rural | | 9 | 21 | Student | Married | Rural | | 10 | 26 | Student-employment | Married | Rural | | 11 | 28 | Factory worker | Separated | Urban | | 12 | 20 | Factory worker | Married | Urban | | 13 | 34 | Factory worker | Married | Urban | | 14 | 31 | Factory worker | Married | Urban | | 15 | 24 | Working in bank | Married | Urban | |
| --- | --- | --- | --- | --- | --- | --- | --- | --- | --- | --- | --- | --- | --- | --- | --- | --- | --- | --- | --- | --- | --- | --- | --- | --- | --- | --- | --- | --- | --- | --- | --- | --- | --- | --- | --- | --- | --- | --- | --- | --- | --- | --- | --- | --- | --- | --- | --- | --- | --- | --- | --- | --- | --- | --- | --- | --- | --- | --- | --- | --- | --- | --- | --- | --- | --- | --- | --- | --- | --- | --- | --- | --- | --- | --- | --- | --- | --- | --- | --- | --- | --- | --- | --- | --- | --- | --- | --- | --- | --- | --- | --- | --- | --- | --- | --- | --- | --- | --- | --- | --- | --- | --- | --- | --- | --- | --- | --- | --- | --- | --- | --- | --- | --- | --- | --- | --- | --- | --- | --- | --- | --- | --- | --- | --- | --- | --- | --- | --- | --- | --- | --- | --- | --- | --- | --- | --- | --- | --- | --- | --- | --- | --- |

**Supplementary material: characteristics of case note review clients and interview and focus group discussion participants**
